# Coupling Between Heterotrophic Nanoflagellates and Bacteria in Fresh Waters: Does Latitude Make a Difference?

**DOI:** 10.3389/fmicb.2016.00114

**Published:** 2016-02-11

**Authors:** Bianca T. Segovia, Carolina D. Domingues, Bianca R. Meira, Fernando M. Lansac-Toha, Paulina Fermani, Fernando Unrein, Lúcia M. Lobão, Fabio Roland, Luiz F. M. Velho, Hugo Sarmento

**Affiliations:** ^1^Núcleo de Pesquisas em Limnologia, Ictiologia e Aquicultura, Universidade Estadual de MaringáMaringá, Brazil; ^2^Departamento de Botânica, Universidade Federal do Rio de JaneiroRio de Janeiro, Brazil; ^3^Instituto Tecnológico de Chascomús, Universidad Nacional de San MartínChascomús, Argentina; ^4^Departamento de Ecologia, Universidade Federal de Juiz de ForaJuiz de Fora, Brazil; ^5^Departamento de Hidrobiologia, Universidade Federal de São CarlosSão Carlos, Brazil

**Keywords:** bacterioplankton, cladocera, protist, predation, latitude

## Abstract

Recent studies reported comparatively lower heterotrophic bacteria (HB) abundances in tropical regions, indicating that factors involved in bacterial losses could be more relevant in the tropics. Heterotrophic nanoflagellates (HNF) are considered the main predators of HB in aquatic ecosystems, and one should expect higher abundances in the tropics because of differences in the food web configuration (absence of large daphnids). However, there are no comprehensive studies comparing HB and HNF abundances in a latitudinal gradient. We hypothesized that HB abundance would be lower in the tropics because HNF abundance would be higher, resulting in a tighter HNF–HB coupling. To test this hypothesis, we compiled a large dataset of HB and HNF abundances from tropical and temperate freshwater environments. We found that both HB and HNF abundances were lower in the tropical region, and that HNF-HB coupling does not differ between temperate and tropical regions. The lower HNF abundance and lack of coupling may be explained by a strong top-down control on HNF and/or their herbivory preference. Besides, no relationship was found between bacterial specific growth rate and either chlorophyll-*a* and HB abundance, indicating that bacterial losses may have an important role in tropical freshwaters. Thus, we found that HNF is likely not the main controllers of HB abundance, and that grazing by ciliates and cladocerans, together with the physiological effects of higher temperatures, may explain the high bacterial loss rates in the tropics.

## Introduction

Inland aquatic ecosystems play a relevant role in the global carbon cycle (Cole et al., 2007; Tranvik et al., 2009; Raymond et al., 2013). Low latitude freshwaters, particularly wetlands, represent a high percentage of global CO_2_ evasion to the atmosphere compared to colder counterparts located in temperate regions (Marotta et al., 2009; Aufdenkampe et al., 2011; Barros et al., 2011; Abril et al., 2014; Borges et al., 2015). The disproportional importance of tropical fresh waters in CO_2_ net diffusion would be due to the high input of organic terrestrial carbon and further microbial heterotrophic respiration (Cole et al., 1994; del Giorgio et al., 1999), together with the higher temperatures (Kosten et al., 2010). In fact, bacterial biomass and production has been related to CO_2_ lake concentrations (Tadonléké et al., 2012; Fontes et al., 2013), evidencing the importance of bacterioplankton in CO_2_ emission dynamics. Thus, it is essential to identify the patterns and drivers of bacterial abundance, production and respiration across latitudinal gradients.

In this way, recent studies pointed out that, despite the slightly higher bacterial production in lower latitudes (Amado et al., 2013), the bacterial abundance found in those regions is lower, compared to temperate environments (Roland et al., 2010; Sarmento, 2012). This indicates that factors involved in bacterial loss would be more important in the tropics, since bacterial biomass does not seem to increase with increasing bacterial production in similar rates in both regions (Billen et al., 1990). The low bacteria:chlorophyll*-a* ratios found in warm waters suggest that grazing might be an important mechanism limiting bacterial abundance (Sarmento et al., 2008; Roland et al., 2010; Özen et al., 2013). These differences in HB abundance at different latitudes have been attributed, at least in part, to a higher top–down control of rotifers, ciliates, and nanoflagellates in warmer regions (Roland et al., 2010; Sarmento et al., 2010; Sarmento, 2012; Vázquez-Domínguez et al., 2012; Amado et al., 2013).

Because heterotrophic nanoflagellates (HNF) are considered the main responsible for channeling bacterial production to higher trophic levels (Fenchel, 1982; Sanders et al., 1989, 1992; Berninger et al., 1991), one should expect a higher top–down control on bacteria by the HNF in the tropics. Factors known to exert an influence on the predator–prey relationship between HNF and bacteria, such as temperature, bacterial, and HNF abundance (Peters, 1994; Vaqué et al., 1994; Gasol et al., 2002), vary widely with latitude. As temperature alters metabolic rates, it also influences all the other factors above cited, as predicted by the metabolic theory of ecology (MTE; Brown et al., 2004), which might also provide some insights on differences of microbial metabolic rates and trophic interactions between tropical and temperate regions.

The cornerstone of MTE is that metabolic rates, including grazing rates (Sarmento et al., 2010) and population growth rates (Savage et al., 2004), increase exponentially with temperature (Brown et al., 2004). For instance, bacterial abundance and production is thought to increase with increasing temperatures (White et al., 1991). However, the effects of temperature are not always straightforward, and increased temperatures may actually lead to a decrease in the abundance of the organisms, because the increased metabolic cost per individual means that a given supply of energy will support a smaller number of individuals (Brown et al., 2004; Savage et al., 2004; Sarmento et al., 2010). Yet, this assumption does not consider the effects of trophic interactions. For example, Jiang and Morin (2004) found that competition between the populations of two protists changed the outcome of temperature effects on their abundances, when compared with the isolated temperature effect on those populations. Also, Vasseur and McCann (2005) model states that temperature alone would not affect resource density in the absence of predators, implying that the effects of trophic interactions should also be taken into account.

Temperature has also been positively correlated with feeding rates, thereupon protist grazing rates on bacteria are expected to be higher with raised temperatures, since more food is required to fulfill their energy demand (Peters, 1994; Vaqué et al., 1994; Sarmento et al., 2010). Considering that tropical regions experience elevated temperatures throughout the year, bacteria might suffer a higher predation pressure, so that a larger proportion of bacterial production is taken by grazers (Sarmento et al., 2010), outbalancing bacterial growth stimulation by temperature. Indeed, in the few studies available for tropical region, HNF grazing on bacteria was found to be relatively high (Pirlot et al., 2007; Tarbe et al., 2011).

It is believed that HNF abundance in warm environments should be higher than in colder ones, owing to consistent differences in the food web structure along the latitudinal gradient (Sarmento, 2012; Özen et al., 2013). This is because in temperate environments there is a typical prevalence of large-bodied cladocerans, which are able to suppress the abundance of HNF (Gasol et al., 1995; Jürgens and Stolpe, 1995; Kalinowska et al., 2015). Actually, the predation pressure of *Daphnia* on HNF was found to result in a lack of coupling between HNF-bacteria in temperate systems, highlighting zooplankton as crucial regulators of bacterial abundance (Gasol and Vaqué, 1993; Jürgens et al., 1994). Meanwhile in the tropics, both temperature (Havens et al., 2015) and the high predation pressure exerted by the juvenile fishes, which are almost permanently present due to fish reproduction throughout the year (Fernando, 1994; Lazzaro, 1997; Iglesias et al., 2011), favor the development of small-bodied zooplankton. Those, in turn, would not be as efficient in reducing microbial abundances as their relatives of the temperate regions, thus the assumed greater HNF abundance would account for a tighter coupling between bacteria and HNF in tropical environments (Sarmento, 2012). Accordingly, elevated temperatures increasing microbial metabolism, along with the higher abundance of HNF and lower abundance of bacteria, all concur to the idea that HNF-bacteria coupling should differ across latitudinal gradients, being stronger in the tropics.

The aim of this study was to compare HNF and HB abundances in different latitudes (temperate vs tropical), as well as the HNF–HB coupling. Taking into account that HNF grazing pressure is thought to be the main explanation for lower bacterial abundance in tropical regions, and that the lack of HNF–bacterial coupling seems to be a widespread phenomenon in the temperate ones, we hypothesized that, in the tropics, (i) HB abundance would be lower, because (ii) HNF abundance would be higher, and consequently (iii) HNF–HB coupling would be stronger. We also investigated the importance of other predators and resources (i.e., chlorophyll-*a*) in explaining bacterial abundance in tropical environments. In order to test these hypotheses, we compiled a large dataset of HB and HNF abundances from tropical and temperate freshwater environments and compared their abundances and the HNF-HB coupling, besides exploring other possible causes involved in bacterial losses in the tropics.

## Materials and Methods

### Data Compilation

The dataset consists of 1047 observations of heterotrophic bacteria (HB) and HNF abundances from the literature in both tropical (*N*_trop_ = 381) and temperate (*N*_temp_ = 666) freshwater inland aquatic ecosystems. The data was gathered from some of the vast literature found for temperate environments as well as studies performed so far in the tropics, and encompasses a broad range of environment types, including shallow lakes, deep lakes, and reservoirs of various trophic status (**Table 1**). We also used abundance data of ciliates, rotifers, cladocerans, and copepods from tropical environments.

**Table 1:** Database from each literature data in tropical and temperate environments used in all analysis. ^∗^To perform the analysis of the relationship between bacterial specific growth rates, chlorophyll-^*a*^, and bacterial abundance, we used a different dataset (see below).

Reference	*N*	HB abundance		HNF abundance	
		Minimum	Maximum	Minimum	Maximum
**Tropical**					
Domingues et al., submitted	46	5.93 × 10^5^	6.17 × 10^6^	1.80 × 10^3^	2.75 × 10^4^
Meira et al., in preparation	21	3.03 × 10^5^	2.50 × 10^6^	6.52 × 10^0^	2.02 × 10^2^
Morana et al., 2014	21	1.82 × 10^6^	4.58 × 10^6^	2.07 × 10^2^	1.11 × 10^3^
Velho et al., in preparation	36	1.46 × 10^5^	7.54 × 10^5^	1.10 × 10^2^	2.35 × 10^3^
Pereira et al., 2014	58	1.18 × 10^6^	8.48 × 10^6^	9.22 × 10^1^	1.56 × 10^4^
Pirlot et al., 2005	21	1.66 × 10^6^	5.63 × 10^6^	2.99 × 10^2^	4.08 × 10^3^
Segovia et al., 2014	72	1.46 × 10^5^	1.26 × 10^6^	1.09 × 10^2^	1.21 × 10^4^
Segovia et al., in preparation	106	4.18 × 10^4^	2.33 × 10^6^	1.78 × 10^1^	1.53 × 10^3^
**Total**	**381**				

**Temperate**					
Bennett et al., 1990	32	2.31 × 10^6^	8.73 × 10^6^	1.12 × 10^3^	5.61 × 10^3^
Berninger et al., 1993	81	7.33 × 10^6^	2.21 × 10^7^	8.07 × 10^3^	7.14 × 10^4^
Bird and Kalff, 1989	12	2.55 × 10^6^	1.34 × 10^7^	2.80 × 10^2^	6.20 × 10^3^
Bloem and Bär-Gilissen, 1989	34	4.00 × 10^6^	1.00 × 10^7^	2.00 × 10^2^	3.40 × 10^4^
Bloem et al., 1989	12	5.42 × 10^6^	1.45 × 10^7^	5.40 × 10^2^	1.05 × 10^4^
Christoffersen et al., 1990	10	4.13 × 10^6^	5.91 × 10^6^	8.75 × 10^1^	1.08 × 10^3^
Fermani et al., 2013^∗^	41	2.34 × 10^7^	1.08 × 10^8^	9.40 × 10^3^	1.12 × 10^5^
Fermani et al., 2015^∗^	36	1.39 × 10^6^	2.87 × 10^8^	1.47 × 10^2^	3.89 × 10^5^
Finlay et al., 1988	6	8.70 × 10^6^	2.10 × 10^7^	5.00 × 10^4^	1.80 × 10^5^
Güde, 1986	7	4.10 × 10^6^	9.40 × 10^6^	2.30 × 10^3^	7.20 × 10^3^
Güde, 1988	9	3.80 × 10^6^	9.95 × 10^6^	1.40 × 10^2^	7.67 × 10^3^
Jürgens and Güde, 1991	19	4.10 × 10^6^	1.24 × 10^7^	1.40 × 10^3^	2.50 × 10^4^
Jürgens and Jeppesen, 2000	10	4.76 × 10^6^	1.56 × 10^7^	2.29 × 10^3^	1.29 × 10^4^
Munawar and Weisse, 1989	72	3.90 × 10^5^	3.35 × 10^6^	4.40 × 10^2^	5.79 × 10^3^
Nakano et al., 1998	16	1.23 × 10^7^	4.87 × 10^7^	3.06 × 10^3^	1.42 × 10^5^
Pace et al., 1990	5	3.10 × 10^6^	7.83 × 10^6^	4.40 × 10^2^	1.05 × 10^3^
Pick and Caron, 1987	22	6.90 × 10^5^	6.20 × 10^6^	4.92 × 10^2^	6.65 × 10^3^
Šimek and Fuksa, 1989	12	1.98 × 10^6^	4.89 × 10^6^	9.20 × 10^1^	1.39 × 10^3^
Šimek et al., 1990	17	1.34 × 10^6^	3.99 × 10^6^	8.60 × 10^1^	1.29 × 10^3^
Šimek et al., 1997	32	2.05 × 10^6^	4.60 × 10^6^	1.35 × 10^3^	4.45 × 10^3^
Sommaruga, 1995	36	1.70 × 10^6^	2.03 × 10^7^	1.14 × 10^3^	2.97 × 10^4^
Vaqué and Pace, 1992	64	2.90 × 10^6^	8.76 × 10^6^	1.69 × 10^2^	1.92 × 10^3^
Weisse, 1990	24	5.69 × 10^5^	6.56 × 10^6^	5.40 × 10^2^	8.11 × 10^3^
Weisse, 1991	103	4.21 × 10^5^	7.99 × 10^6^	3.14 × 10^2^	7.97 × 10^3^
Wieltschnig et al., 2001	31	2.91 × 10^6^	6.66 × 10^6^	5.59 × 10^2^	2.34 × 10^3^
**Total**	**743**				

^∗^*Data from those references were considered outliers and were not used in our analysis.*

### Data Analysis

#### HB and HNF Abundance and Relationship

To test whether HB and HNF abundances differ among tropical and temperate freshwater environments, we used non-parametric Mann–Whitney Rank Sum test. In addition, we also performed non-parametric Mann–Whitney Rank Sum test to compare median values of HB:HNF ratios between tropical and temperate environments. To examine the relationship between HNF and HB on tropical and temperate datasets, we performed model II linear regression using the major axis (MA) method (Legendre, 2014), and verified the normal distribution of the log-transformed data. We compared the slopes and intercepts for both regions using the “ma” function of the “smatr” package (Warton et al., 2012), that tests hypotheses about slope or elevation (“elev.test”) based on confidence intervals comparison.

#### Bacterial Specific Growth Rate (SGR) Relationship with Chlorophyll-*a* and Bacterial Abundance

We used a dataset comprehending several tropical environments sampled in different seasons (Lobão et al., in preparation) to verify if bacterial SGR was more related to resources or predators. We estimated bacterial SGR using the equation proposed by Kirchman (2002): SGR = P/B, where *P* = bacterial production (μgC L^–1^ h^–1^) and B = bacterial biomass (μgC L^–1^). We performed linear regressions to test the relationship between SGR and HB abundance, which might provide some hints about the factors controlling their abundance. The rationale is that, considering the density-dependent logistic growth of bacteria, SGR is low when bacterial abundance is reaching the carrying capacity, meaning that they are limited by resource availability. Hence, a negative relationship between SGR and abundance indicates bottom-up control. Conversely, SGR is high when bacterial abundance is far from reaching the carrying capacity. Thus, the lack of relationship between SGR and abundance indicates top-down control, so that predators could be consuming bacteria at rates equal to or higher than their production (Wright and Coffin, 1984; Gasol et al., 2002).

#### Impact of Other Communities on HB and HNF Abundance

We examined the effects of potential predators on HB and HNF in the tropical region. We considered the abundances of HB and HNF as response variables separately, and performed multiple regressions for each one. For HB, we used the abundance of the predators HNF, ciliates, rotifers and cladocerans as explanatory variables, excluding copepods, which have a very low capture efficiency of picoplankton (Wilson, 1973; Finlay and Roff, 2004; Sommer and Sommer, 2006). For HNF, we used the abundance of the predators known to exploit them as food, such as ciliates, rotifers, cladocerans, and copepods.

Data was log-transformed and all analyses were performed in R Development Core Team (2013) using the libraries “vegan” (Oksanen et al., 2015), “lmodel2” (Legendre, 2014), and “smatr” (Warton et al., 2012). Figures were made on SigmaPlot v.12 software (Systat Softare Inc.).

## Results

At first, we considered all data we gathered from the literature in our analyses. However, some of the studies performed in highly eutrophic environments have found extreme values of HB and HNF abundance, never reported before on the literature (i.e., Fermani et al., 2013, 2015). As we did not found any equivalent conditions in the tropical dataset, and since we noticed that the data from those studies were outliers, we decided to disregard those values from all our analyses. In this way, we maintained a similar distribution of points among trophic states in the temperate (oligotrophic: 16%, mesotrophic: 50%, eutrophic: 34%) and tropical (oligotrophic: 19%, mesotrophic: 45%, eutrophic: 36%) regions. Nevertheless, we show them in the regression figure (**Figure 3**) for comparison purposes.

### HB and HNF Abundance

**FIGURE 1:**
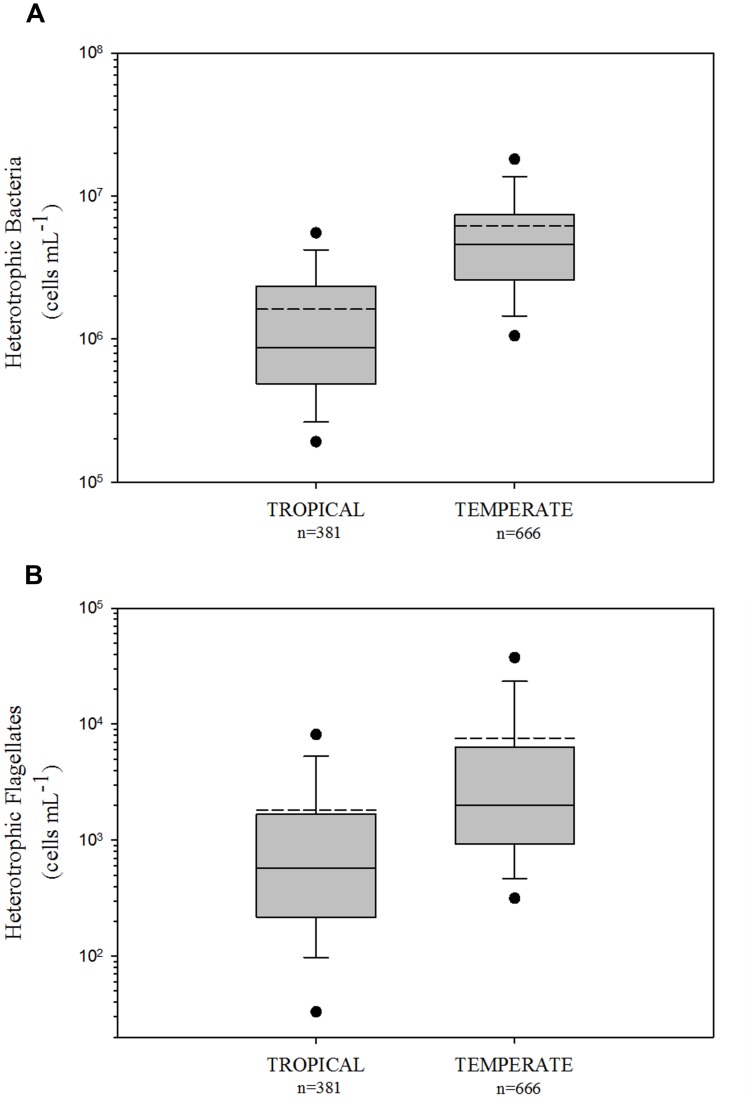
**Comparison of heterotrophic bacteria **(A)** and heterotrophic flagellates **(B)** among tropical and temperate freshwater environments.** The central full line indicates the median value, the dotted line indicates the arithmetic mean value, the boxes indicate the lower and upper quartiles, the vertical lines indicate the 10th and 90th percentiles, and the dots represent the 5th and 95th percentiles. Tropical and temperate data were significantly different (non-parametric Mann-Whitney Rank Sum test) in the two variables (HB and HNF with *p* < 0.001, see text for details).

Comparing HB and HNF abundance in tropical and temperate freshwater environments, we found higher values in the temperate region for both HB (logHB: *p* < 0.001, Mann–Whitney *U* Statistic = 218964.5; **Figure 1A**) and HNF (logHNF: *p* < 0.001, Mann–Whitney *U* Statistic = 189579; **Figure 1B**) communities.

### HNF–HB Relationship

**FIGURE 2:**
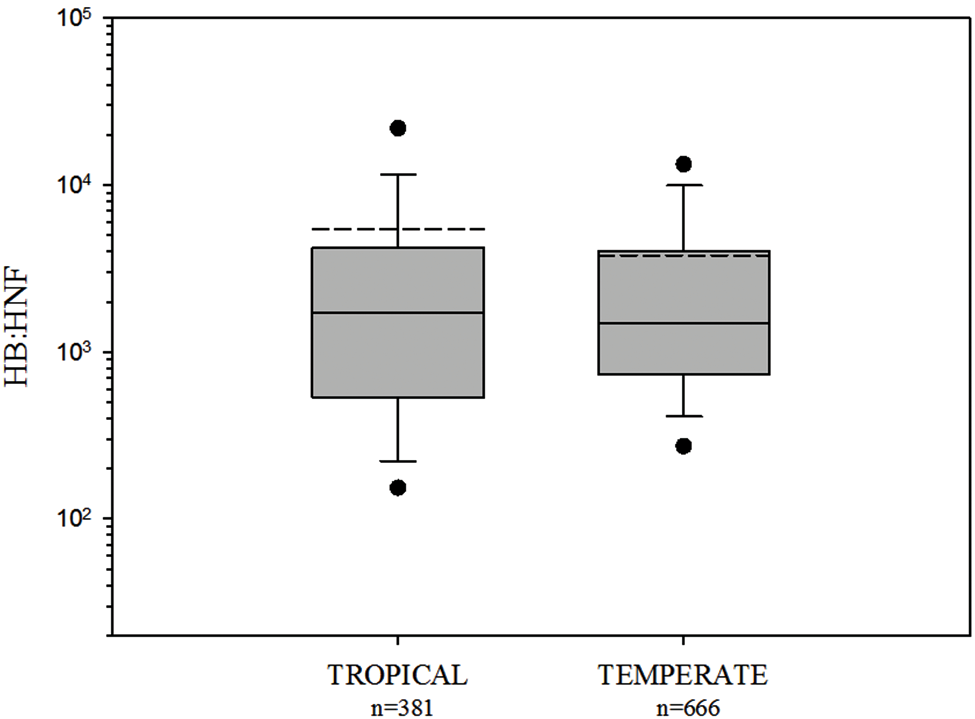
**Comparison of HB:HNF ratios among tropical and temperate freshwater environments.** The central full line indicates the median value, the dotted line indicates the arithmetic mean value, the boxes indicate the lower and upper quartiles, the vertical lines indicate the 10th and 90th percentiles, and the dots represent the 5th and 95th percentiles. HB:HNF ratios were not significantly different between tropical and temperate environments (see text).

HB:HNF ratios were not significantly different between tropical and temperate environments (HB:HNF: *p* = 0.3049, Mann–Whitney *U* Statistic = 131703; **Figure 2**).

**FIGURE 3:**
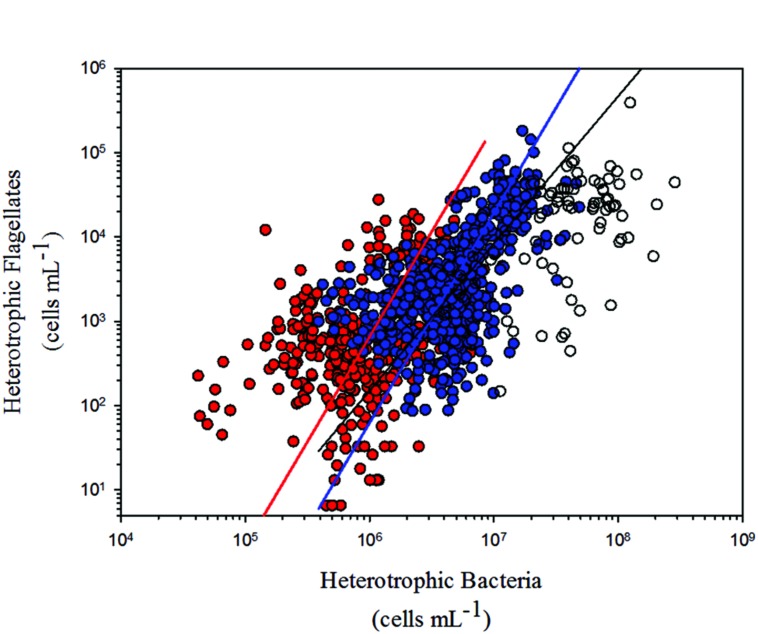
**Model II linear regressions between HNF and HB for tropical (red dots and red line; *r*^2^= 0.14; *p* < 0.0001) and temperate (blue dots and blue line; *r*^2^= 0.30; *p* < 0.0001) freshwater environments (see **Table 2** for confidence intervals).** Outliers disregarded from our analyses (blank dots) and model II linear regressions for temperate freshwater environments including those outliers (black line) are also shown (see Materials and Methods).

We found a significant positive relationship between HNF and HB for tropical and temperate regions. Comparing the regression models from both regions, we found no significant differences between the slopes, besides no differences in the confidence intervals for the intercepts (**Table 2**; **Figure 3**).

**Table 2:** Model II Linear Regression parameters between HNF and HB for tropical and temperate regions.

LogHNF vs. LogHB	Slope	95% (ci)	Intercept	95% (ci)	*n*	*r*^2^	*p*
Tropical	2.49	(1.98:3.29)	–12.12	(–16.92:–9.04)	381	0.14	<0.0001
Temperate	2.48	(2.22:2.81)	–13.13	(–15.28:–11.38)	666	0.3	<0.0001

### Factors Controlling HB and HNF Abundances

**FIGURE 4:**
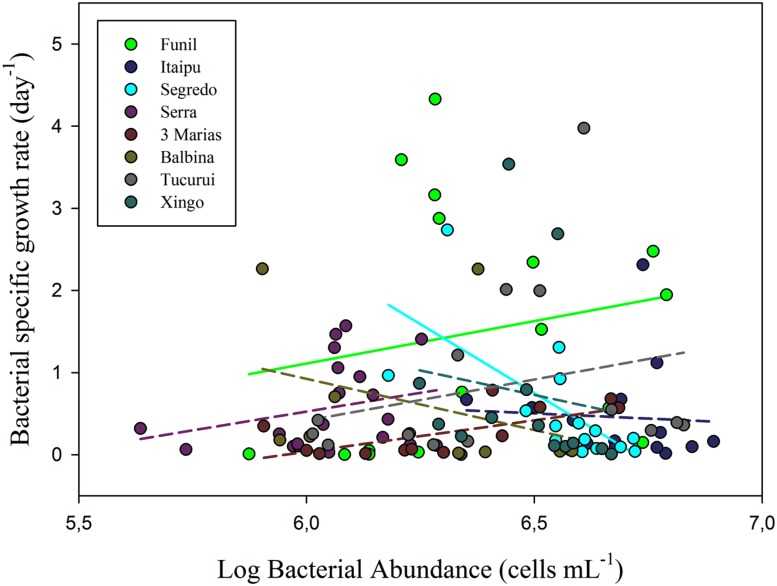
**Relationship between bacterial SGR and HB abundance in several tropical freshwater environments.** Most linear regressions (six out of eight) were non-significant, suggesting regulation by predation. One regression was positive (β = 0.13; *r*^2^ = 0.30) and only one was negative suggesting resource limitation, although not a very strong one (β = –0.18; *r*^2^ = 0.49).

Linear regressions between SGR and HB abundance (**Figure 4**) were non-significant in most tropical systems (six out of eight systems), pointing toward a regulation of bacterial numbers by predation for most systems. Taking all systems together, this relationship was not significant either.

We performed multiple regressions to evaluate the effects of potential predators on HB abundance. The regression model explained 28% of the variation in HB abundance of the tropical data and included the abundances of HNF, ciliates, rotifers and cladocerans (**Table 3**). The standardized regression coefficients of both HNF and rotifers were positive, thus an increase in HNF and rotifer abundance was associated with an increase in HB abundance, suggesting a bottom-up effect. As for the ciliates and cladocerans, we found a negative relationship, suggesting a top down effect, since an increase in ciliate and cladoceran abundance was associated with a reduction in HB abundance.

**Table 3:** Regression analyses for HB and HNF abundance of the tropical region.

	Models		β (±SE)				
		*r*^2^	HNF	Cili	Rot	Clad	Cop
HB	HNF, Cili, Rot, Clad	0.28	0.55 (±0.05)	**– 0.36** (±0.05)	0.31 ( ± 0.05)	**– 0.13** (±0.06)	–
HNF	Cili, Clad	0.32	–	0.60 ( ± 0.05)		**– 0.17** (±0.06)	–

β, *standard regression coefficient; SE, standard error; HB, heterotrophic bacteria; HNF, heterotrophic nanoflagellates; Cili, ciliates; Rot, rotifers; Clad, cladocerans; Cop, copepods. Bold values are the negative* β *values.*

The best multiple regression model for HNF abundance included only ciliates and cladocerans and explained 32% of the HNF abundance variation. The standard regression coefficient of ciliates was positive, indicating a simultaneously increase in both variables. As for the cladocerans, we found a negative relationship, indicating a top–down effect of this group on HNF (**Table 3**).

## Discussion

We compiled for the first time a consistent HNF and HB abundance database for tropical freshwaters, and compared the abundances of those communities with the ones from the temperate environments, as well as explored probable causes of lower bacterial abundance in the tropics. We found that both HNF and HB abundances were lower in the tropics and that there is no difference in the HNF-HB coupling between those regions. Besides, HB abundances were apparently more regulated by predation, especially from ciliates and cladocerans.

### Weak Evidence of Resource Limitation

Evidence found in the literature suggests that bacterial growth dependence on phytoplankton derived dissolved organic carbon (DOC) supply might not always be that relevant in low latitudes. Although there is evidence that phytoplankton derived DOC would be important for the bacterioplankton of large African tropical lakes (Stenuite et al., 2009; Morana et al., 2014), low HB:phytoplankton biomass ratios have been found (Sarmento et al., 2008). In a comparative analysis using different types of Brazilian freshwater ecosystems, Roland et al. (2010) found a much weaker HB:chlorophyll-*a* correlation in tropical when compared to the non-tropical environments. In this way, the bacteria–phytoplankton uncoupling seems to be a recurrent situation in south-American lowland lakes (e.g., Carvalho et al., 2003; Gocke et al., 2004; Rejas et al., 2005; Petrucio et al., 2006; Teixeira et al., 2011; Almeida et al., 2015), which are generally smaller and shallower, comparing to the East-African Great Lakes. White et al. (1991) reported a rather weak correlation between SGR and chlorophyll-*a* in freshwaters, and suggested that variations in the importance of grazing pressure may have contributed to this finding.

However, as allochthonous DOC may also constitute an important resource for HB (Tranvik, 1992), a regulation of HB by those carbon sources could also explain the weak dependency of bacteria on phytoplankton. Unfortunately, we do not have data concerning those variables, which would allow us to elucidate this point. Nonetheless, our results of non-significant relationships between bacterial SGR and HB abundance in most of the tropical systems analyzed (**Figure 4**), reinforce the idea that predation might be more relevant than resource limitation, whatever that resource could be. If HB abundance and SGR were not related, grazing was likely consuming HB at such a rate that it was limited by a small range of possible growth rates (Wright and Coffin, 1984; Gasol et al., 2002). Thus, we could infer that resource limitation was not likely to restrain HB abundance in most tropical freshwater environments, and that a top-down control might prevail in these systems.

### HNF Abundance is Also Lower in Tropical Environments

The assumption of a higher abundance of HNF in tropical, relative to temperate environments, was not corroborated in our study. Although large-bodied cladocerans are relatively low abundant in the tropics, the typical small bodied cladocerans, seem to exert a strong predation pressure on HNF, as evidenced by the negative standard coefficient multiple regression model (**Table 3**).

The impact of small-bodied cladocerans on HNF is somewhat unexpected, since in the tropics there is usually a smaller proportion of Daphniidae, which is replaced by Bosminids, Sidids, and Moinids (Dumont, 1994; Elmoor-Loureiro, 2000). However, the influence of cladocerans on the abundance of HNF was already verified in the bottom layer a tropical floodplain lake where those predators were more abundant, specially represented by *Bosmina hagmanni* and *Ceriodaphnia cornuta* (Segovia et al., 2014). In fact, the small-bodied cladocerans *Bosmina*, *Ceriodaphnia*, and *Diaphanosoma* were found to achieve higher weight-specific clearance rates on HNF than that of *Daphnia* species (Jürgens et al., 1996). Specifically, *Bosmina* have a particular foraging mode, different from filter-feeding, which allows certain selectivity and consequently more efficient removal of small flagellates compared to *Daphnia* (DeMott and Kerfoot, 1982), even at low food concentrations (DeMott, 1982). Thus, even though Daphnids are recognized as the main responsible for hampering the development of HNF in temperate ecosystems (Pace and Vaqué, 1994; Gasol et al., 1995; Jürgens and Stolpe, 1995), their low abundance in the tropics would not result in a weaker predation pressure of cladocerans on HNF, since other small-bodied cladocerans such as the Bosminids may replace *Daphnia*, in the sense that they would also be able to suppress HNF effectively. As for the ciliates, we found a positive relationship with HNF, indicating that both variables are increasing. It is possible that this could be the result of the control of both HNF and ciliates by variables related to their shared resources and predators (Auer et al., 2004; Segovia et al., 2014; Domingues et al., submitted).

HNF–HB coupling in the tropics does not seem to differ from that of the temperate regions. A top–down control by cladocerans on HNF may be keeping them from reaching the high abundances they presumably would have in the tropics, blurring their effects on bacteria (Gasol and Vaqué, 1993; Gasol, 1994; Wieltschnig et al., 2001; Segovia et al., 2014; Kalinowska et al., 2015). Another possible cause for the lack of HNF–HB coupling is the use of an alternative food resource by the HNF, such as the picophytoplankton (PPP). Herbivory preference by nanoflagellates, rather than bacterivory, was verified in the large tropical Lake Tanganyika (Tarbe et al., 2011). The preference of HNF for PPP was also found in shallow floodplain lakes in the tropical region (Meira et al., in preparation). In addition, the biomass of HNF was negatively related to PPP in tropical reservoirs of different trophic states, pointing out the importance of this interaction on these environments as well (Domingues et al., submitted). To sum up, the lower HNF abundance found, together with the similar HNF-HB coupling, suggests that HNF is probably not related to the lower HB abundance in the tropics.

### Grazing by Ciliates and Cladocerans May Explain the Lower HB Abundance in the Tropics

The variables associated with HB abundance in the tropics were HNF, ciliates, rotifers, and cladocerans. HNF and rotifers were positively related with HB abundance, which means that they are likely feeding on bacteria but are not able to suppress their abundance. On the contrary, both ciliates and cladocerans showed a negative relationship, suggesting a top–down control on HB abundance. As stated before, resource limitation or predation by HNF are unlikely to be the reason why bacterial abundance is lower in the tropics. Thus, the negative effect of both ciliates and cladocerans could be part of the explanation for such a pattern.

Although there is a vast literature relating the prevalence of HNF as the major bacterivores (Fenchel, 1982; Sanders et al., 1989, 1992; Berninger et al., 1991), the relatively higher importance of ciliates as predators of bacteria was also documented. The dominance of ciliates as grazers of bacteria has been reported in occasions where HNF abundance is rather low (Kisand and Zingel, 2000; Tadonléké et al., 2005; Zingel et al., 2007). Also, ciliate community structure in the tropics may differ from that of the temperate regions. It is known that bacterivory is predominant among the small oligotrich ciliates (Stabell, 1996; Šimek et al., 2000), thus perhaps features such as ciliate community composition might be playing a role on the impact of ciliates in tropical environments, where there could be a larger proportion of those bacterivorous taxa. However, more studies are necessary to draw such a conclusion. Another overlooked aspect would be the influence of temperature on the ciliate feeding rates. It has been shown that ciliate feeding rates increase considerably with the raise of temperature (Sherr et al., 1988; Rychert, 2011). Therefore, it is plausible to infer that the higher temperatures of the tropics may be a relevant factor.

Similarly to the impact on HNF, weight-specific filtering rates on bacteria were found to be higher for *Ceriodaphnia* and *Bosmina* than for the large *Daphnia magna* (Porter et al., 1983). Vaqué and Pace (1992) found that lakes dominated by large populations of *Bosmina longirostris* showed even slightly higher maximum values of grazing on bacteria (35 × 10^6^ bacteria L^–1^ h^–1^) than *Daphnia pulex* (30 × 10^6^ bacteria L^–1^ h^–1^), and concluded that, when in large numbers, populations of small cladocerans compensate for the lack of large Daphnids. Thus, if tropical environments are dominated by those small-bodied cladocerans, then their impact on bacterioplankton could be higher than in temperate environments. In addition, a positive relationship has been found between cladoceran filtering rate and temperature (Burns, 1969). For example, Mourelatos and Lacroix (1990) found that at a temperature of 20°C, a *Daphnia* of 0.5 mm size filtered as much as one twice its size but at a 10°C temperature, suggesting that at higher temperatures those small-bodied cladocerans should have an even greater impact. Moreover, a recent study found that pelagic cladocerans significantly explained the variation in bacterial community composition in tropical South American shallow lakes (Souffreau et al., 2015), demonstrating that the predation pressure of those microcrustaceans might also be responsible for changes in bacterial community structure.

**FIGURE 5:**
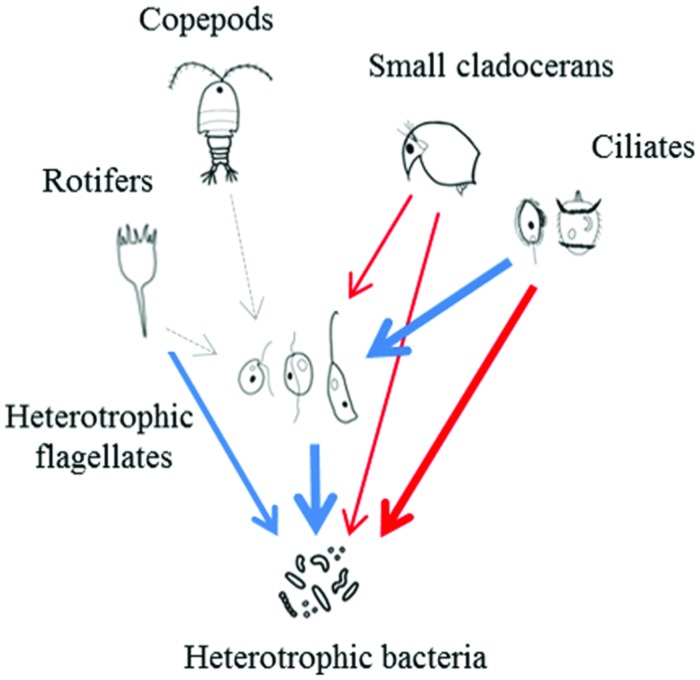
**Schematic representation showing possible impacts of predators and resources on HB and HNF in tropical regions.** Dashed arrows indicate no relationship, blue arrows indicate positive and red arrows indicate negative relationships. The thickness of the arrows is proportional to the strength of the interaction.

Thus, ciliates and small cladocerans seem to play a central role in the pelagic food webs of tropical freshwater environments, and the fundamental differences in the food web structure of freshwater environments in temperate and tropical environments, together with the higher temperatures of the tropical ones, likely dictate the fate of bacterial production (**Figure 5**).

It is worth noting that virus lysis is also recognized as a major source of bacterial losses (Fuhrman and Noble, 1995), however, few studies concerning this topic were performed in the tropics. Low virus-to-bacterium ratios and frequency of visible infected cells were found in Amazonian floodplain lakes (Barros et al., 2010; Almeida et al., 2015) and African lakes (Bettarel et al., 2006). Barros et al. (2010) suggested that these low values could be related to the registered low bacterial abundances, which restrain the rates of encounter between the virus and the bacterial host cell, resulting in a low level of viral predation. As a corollary for this explanation, the comparable lower abundances of bacteria in the tropics should result in lower loss rates by viral attack than in the temperate systems. Nonetheless, relatively high values of virus-to-bacterium ratios were found in tropical reservoirs (Peduzzi and Schiemer, 2004) and in a tropical lake (Araújo and Godinho, 2009). Thus, bacterial mortality caused by virus should be taken into account when studying mechanisms controlling bacterial abundance in tropical freshwaters in the future to elucidate this issue.

## Conclusion

Comparing tropical against temperate data reinforced the previous findings that bacterial abundance is lower in the tropics. Moreover, bacterial specific growth rate was not related to either chlorophyll-*a* and HB abundance, pointing to an important role of bacterial losses in the tropics. Besides, we found that HNF abundance is also lower in the tropics and that HNF-HB coupling is not different across latitudes. A top–down control on HNF and their herbivory preference may help explain the lack of HNF–HB coupling, and suggests that HNF is likely not the main cause for bacterial loss. It is possible that grazing by ciliates and cladocerans play a large role in controlling bacterial abundance in the warmer regions. However, this issue should be more investigated in future studies concerning tropical freshwater environments.

## Author Contributions

HS and BTS conceived and designed the study. CDD, BRM, FMLT, PF, FU and LFMV sampled and analyzed the abundance data of microbial communities from tropical/temperate freshwaters. LML and FR sampled and analyzed the bacterial specific growth rate data from tropical freshwaters. BTS wrote the manuscript with input from all coauthors.

## Conflict of Interest Statement

The authors declare that the research was conducted in the absence of any commercial or financial relationships that could be construed as a potential conflict of interest.
